# First-Principles Investigation of Adsorption and Diffusion of Ions on Pristine, Defective and B-doped Graphene

**DOI:** 10.3390/ma8095297

**Published:** 2015-09-15

**Authors:** Wei Wan, Haidong Wang

**Affiliations:** School of Minerals Processing and Bioengineering, Central South University, Changsha 410083, China; E-Mail: wwhongye@hotmail.com

**Keywords:** first-principles calculation, graphene, defects and B doping, adsorption energy, diffusion barrier, anode materials

## Abstract

We performed first-principles calculations to reveal the possibility of applying pristine, defective, and B-doped graphene in feasible negative electrode materials of ion batteries. It is found that the barriers for ions are too high to diffuse through the original graphene, however the reduced barriers are obtained by introducing defects (single vacancy, double vacancy, Stone–Wales defect) in the graphene. Among the three types of defects, the systems with a double vacancy could provide the lowest barriers of 1.49 and 6.08 eV for Li and Na, respectively. Furthermore, for all kinds of B-doped graphene with the vacancy, the systems with a double vacancy could also provide the lowest adsorption energies and diffusion barriers. Therefore, undoped and B-doped graphene with a double vacancy turn out to be the most promising candidates that can replace pristine graphene for anode materials in ion batteries.

## 1. Introduction

At present, although the rechargeable lithium ion battery (LIB) has been widely used in various types of electronic equipment, it still faces the potential risk of gradually rising cost of lithium [1]. Therefore, the sodium ion battery (SIB), a similar electrochemical system to LIB, has been resurged naturally, because of its lower cost [2,3,4,5,6,7,8,9,10]. However, there are some issues with the anode material for SIBs. Graphite, the successful material for LIBs anode, cannot be applied in SIBs anode, since Na ions hardly intercalate into the graphite layers [11,12,13]. Furthermore, specific capacity of graphite anode (372 mAh/g) is far behind the request of LIBs in the miniaturization of electronic equipment [14,15]. Hence, the key point in the development of LIB and SIB technologies is exploring an efficient anode material [16].

Recently, some results of experiments show that it is possible to use graphene [17,18] and its oxide [19] as the anode materials instead of graphite in LIBs. The specific capacity of graphene anode is 540 mAh/g in LIBs [20]. In addition, a report about reduced graphene oxide (RGO), a suitable anode material in SIBs, can also be found [21]. These results give hope for graphene in competition with other kinds of anode materials [22,23,24,25].

Besides the high capacity, a promising anode material should also be corrosion resistant in organic electrolytes and form a stable solid electrolyte interphase (SEI). In experiment, graphene was prepared to protect the silicon anode in LIBs as a coating, which acted as an artificial SEI [26]. It prevented the silicon anode being exposed to the electrolyte directly, and resulted in a high capacity of 1600 mAh/g. Similar to the case in LIBs, the tailored SEIs of graphene might meet the requirement of anodes in SIBs.

On the one hand, for the practical purpose of using graphene as a protective coating for the anode material in LIBs and SIBs, the barriers should be low enough for Li and Na diffusing through the graphene. On the other hand, according to the following equation, $t=L^{2}/D$, where *t*, *L* and *D* are the ion diffusion time, the diffusion length and the diffusion coefficient, respectively [27]. The current diffusion rate can be further improved by the reduced dimensions of graphene in the same electrochemical cell. Hence, the successful application of graphene is determined by the diffusion rate of Li and Na, through and along the graphene.

There are more and more computational studies focusing on the interaction between ions and graphene now. Some theoretical research investigated the bonding and charge transfer between the adsorbed Li and pristine graphene [18,28,29,30]. However, the defects were observed by transmission electron microscopy (TEM) [31,32] and scanning tunneling microscopy (STM) [33,34] techniques in the graphene structure experimentally. Currently, several density functional theory (DFT) studies showed the adsorption behavior of Li and Na on defective graphene, which exhibited a higher capacity in ionic storage [35,36,37,38,39,40,41,42].

Furthermore, it is said that chemical modification is also an effective way to adjust the performance of graphene [43,44,45,46] in energy applications. The results of electron transport simulation of RGO including C vacancies and the O substitution of edge C atoms show that electrons are localized within 10–40 nm. Liao *et al.* examined mechanisms of charge transfer upon interaction of hydrated protons with a variety of doped graphene nanoribbons, and found that the B, N, and O doped edges all show active proton affinity. In addition, their theory study of charge carrier adsorption onto zigzag edge-shaped graphene nanoribbons (ZGNRs) indicates that a maximum charge loading is achieved with incorporating edge substitution with boron.

Based the above summary of studying on the electrochemical properties of pristine, defective, and chemically modified graphene, two outstanding issues still exist: how defects and chemical doping affect the diffusion of Li and Na on graphene, and what are the differences of the diffusion between the two of them. To answer these questions in detail, in the present study, the first-principles calculations have been executed to thoroughly explore the Li and Na diffusion through the defected graphene including vacancies and Stone–Wales defects. Furthermore, because the boron doping keeps the planar structure of graphene without too much deformation [41], boron-doped graphene has been considered as well. The calculations of barriers through the defected doped graphene have been carried out to reveal the effect of boron doping on manipulating the diffusion rate of Li and Na. These results will contribute to understanding the role of defect types and the concentrations of boron, on Li and Na adsorption and diffusion, thus providing the useful defected doped graphene for the anode material or artificial SEI.

## 2. Theoretical Method

In this work, first-principles calculations were implemented in CASTEP (Cambridge Sequential Total Energy Package, CASTEP Developers’ Group, Cambridge, UK) code [47], which employs pseudopotentials [48] to describe electron-ion interactions and represents electronic wave functions using a plane-wave basis set, based on density functional theory (DFT) [49]. The exchange-correlation energy of many-electron systems is described by the generalized gradient approximation (GGA) with the parametrization of Perdew–Burke–Ernzerhof (PBE) [50] method. The energy cutoffs of 400–800 eV for the plane wave expansion and k-point set 4 × 4 × 1, 6 × 6 × 1 and 8 × 8 × 1 sampled by Monkhorst–Pack method were tested to make sure the total energy is converged at the 1 meV/atom level. The results of test show that the energy cutoff of 650 eV and the k-point grid of 6 × 6 ×1 for the Brillouin zones of the 32-atom supercell is sufficient.

**Figure 1:**
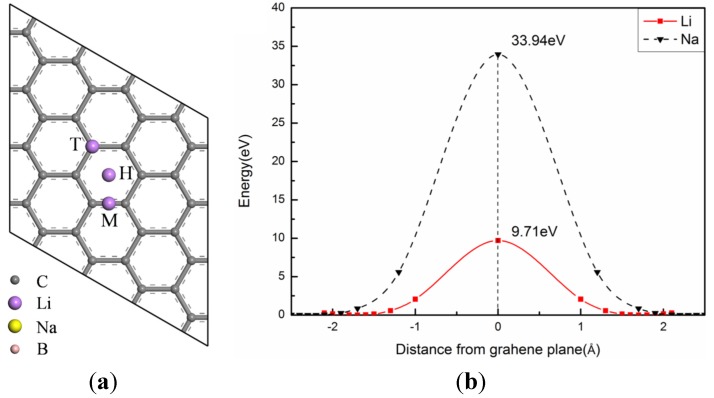
(**a**) Top view of 4 × 4 pristine graphene supercell with ion adsorption on three symmetrically non-equivalent sites (H, T, M); (**b**) Diffusion barriers for Li and Na to penetrate the pristine graphene via the center of the carbon hexagon.

The primitive unit cell of graphene with the experimental lattice constant of 2.46 Å was built by placing the two C atoms on two-dimensional honeycomb lattice with a hexagonal structure. Based on the primitive cell, the supercell consists of 4 × 4 primitive cells, as shown in Figure 1a. The distance of 20 Å was set to avoid the spurious coupling effect between adjacent graphene layers. Then, the single vacancy (SV), double vacancy (DV) and Stone–Wales (SW) defect were constructed in 4 × 4 supercell. Li and Na were kept initially above the plane of the graphene, and then the structures were fully optimized, the relaxation would stop if all forces were smaller than 0.01 eV/Å. The change of lattice constants is very small and can be ignored. Ignored spin polarization approach is applicative in this paper. The corresponding plots for the comparison of both methods (with and without spin polarized calculations) were discussed in Figure S1, Tables S1 and S2 in the Supplementary Information.

## 3. Results and Discussion

### 3.1. Adsorption and Diffusion of Ions on Pristine Graphene

Li and Na are located initially at the position 2.0 Å and 2.5 Å from the graphene plane, respectively. To find the most stable position, three adsorption sites with high symmetry are considered by optimizing the structures: in the center of a carbon hexagon (H), on the top of a carbon atom (T), at the midpoint of a C-C bond (M), as shown in Figure 1a. The adsorption energy (*E_ad_*) have been calculated by the following equation

(1) $$E_{ad}=E_{sy+ion}-E_{sy}-E_{ion}$$

where *E_sy + ion_*, *E_sy_* and *E_ion_* are the total energies of the graphene system with a ion(Li or Na), the graphene system and a ion, respectively. For both Li and Na, the results of the three sites after relaxation show that the H site is the most stable position with the lowest value of +0.49 and +0.61 eV separately. The positive adsorption energies indicate that the adsorption of ions on pristine graphene is unstable energetically. Therefore, the clustering of ions is finite and the diffusion of ions is difficult on the graphene. Furthermore, after relaxation, the most stable position for Li is 1.71 Å away from the plane of the graphene above the center of the hexagon, as well as 2.30 Å for Na.

Next, we calculate the energy barrier for ions to diffuse through the graphene plane. In order to save computational time, the barriers were calculated without relaxing the intermediate structures. This simplified method turns out to be a good approximation as relaxation plays a very small role in bringing down the barrier height. We validate our approach for a few representative systems against a complete linear and quadratic synchronous transit (LST/QST) transition state (TS) search algorithm in CASTEP [51]. The barrier height obtained using both methods are in close agreement (relative error < 5%), as shown in Table 1. The corresponding plots for the LST/QST methods are presented in Figure S2 in the Supplementary Information. According to the results of test calculations, the barriers for ions to diffuse through the graphene were estimated by moving the ion from one site gradually to the reverse site on the same hexagon. During those processes, all the atoms in the graphene layer were fixed, and the energy of each step was calculated separately in the diffusion path. The step of minimum energy was regarded as the zero of the energy, and the diffusion barrier height was approximatively obtained by the energy difference between the maximum energy and minimum energy step. A part of the original data of distances and energies for each step are provided in Table S3 in the Supplementary Information. As shown in Figure 1b, Li and Na must, respectively, overcome higher diffusion barriers of 9.71 and 33.94 eV to penetrating the graphene at the ambient condition for all practical purposes. The reason for this result is the Coulomb repulsion increases with the reduced distance between the ion and the nearest neighbor C atoms.

**Table 1:** Comparison of energy barriers (*E_b_*) obtained by simplified and complete linear and quadratic synchronous transit (LST/QST) method for a few representative systems.

System	*E_b_* by Simplified Method (eV)	*E_b_* by LST/QST (eV)	Error (%)
Gr-Li	9.71	9.74	0.31
Gr-Na	33.94	33.96	0.06
Gr-DV-Li	1.49	1.54	3.25
Gr-DV-Na	6.08	6.14	0.98
Gr-DV-2B(c)-Li	2.25	2.36	4.66
Gr-DV-2B(c)-Na	7.73	7.79	0.77

### 3.2. Adsorption and Diffusion of Ions on Defected Graphene

**Figure 2:**
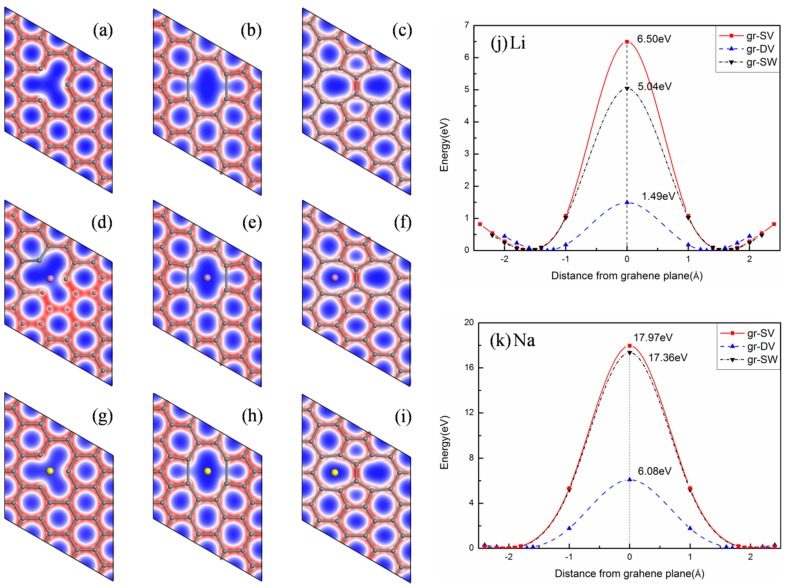
Distribution of the total electronic charge density for defected graphene: (**a**) single vacancy (SV); (**b**) double vacancy (DV); (**c**) Stone–Wales (SW); (**d**) SV with Li; (**e**) DV with Li; (**f**) SW with Li; (**g**) SV with Na; (**h**) DV with Na; (**i**) SW with Na; (**j**) and (**k**) corresponding diffusion barriers for Li and Na.

We consider the possibility of the ion diffusion through the defected graphene, including single vacancy (SV) [33], double vacancy (DV) [52,53] and Stone–Wales (SW) defects [54]. As shown in Figure 2a–c, these defects can be achieved, respectively, by removing a carbon atom, removing C-C dimers, rotating one C-C bond by 90° with respect to the midpoint of the bond from pristine graphene. The barriers for Li (Na) to diffuse through SV (6.50 (17.36) eV), DV (1.49 (6.08) eV) and SW (5.04 (17.97) eV) are observably lower than that in original graphene, as illustrated in Figure 2j,k. It demonstrates that although the barriers are larger for SV and SW defects, for DV defect, it is low enough to meet the requirements toobtain faster Li kinetics in the typical charge–discharge process. It can also be found that the diffusion barrier is determined by the size of the open space formed by the defects. Therefore, both Li and Na can easily diffuse through the graphene via the large octagon spaces formed by DV defect, as shown in Figure 2e,h. Furthermore, as shown in Table 2, the adsorption energies of Li (Na) for SV, DV, and SW defects are −1.12 (−0.58) eV, −0.59 (−0.53) eV and 0.17 (0.27) eV, respectively. The positive adsorption energies of ions for SW defect denote that the binding is not so strong between ions and the graphene. However, all of the adsorption energies for SV and DV defects are negative and therefore, thermodynamically, they all should contribute to trapping ions and avoiding the clustering of Li and Na. When trapping one Li, the interaction between Li and SV lead to the buckling of the graphene plane, as illustrated in Figure 2d. It makes the adsorption energy of Li become stronger. In summary, the graphene with DV is found to be a proper structure for ions diffusion due to its lower diffusion barriers and adsorption energies.

**Table 2:** Adsorption energy (*E_ad_*) of ions, distance of ions from the graphene sheet (*d*) and diffusion barrier (*E_b_*) for the pristine and defected systems investigated.

System	Li			Na		
	*E_ad_* (eV)	*d* (Å)	*E_b_* (eV)	*E_ad_* (eV)	*d* (Å)	*E_b_* (eV)
Pristine graphene (H)	0.49	1.71	9.71	0.61	2.30	33.94
Pristine graphene (T)	0.83	1.94	/	0.70	2.50	/
Pristine graphene (M)	0.81	1.94	/	0.69	2.50	/
Gr-SV	−1.12	1.74	6.50	−0.58	2.07	17.97
Gr-DV	−0.59	1.31	1.49	−0.53	1.90	6.08
Gr-SW	0.17	1.60	5.04	0.27	2.10	17.36

### 3.3. Adsorption and Diffusion of Ions on B-doped Graphene

It is said that chemical doping is an effective way to adjust the performance of graphene [24,55]. Recently, significant experimental progress in B-doped graphene has been made. It reported that the capacity of B-doped graphene reached 235 mAh/g [56]. Furthermore, B-doping greatly decreases the adsorption energy in SIB system, and the capacity of Na_1_(BC_31_) is about 70 mAh/g [57]. We also note that theoretical studies have focused on the lithium and sodium storage on B-doped graphene by calculating the specific capacity [41,58]. However, the effect of B-doping on the diffusion barrier of Li and Na have not been discussed, which is very important for the negative electrode material.

**Figure 3:**
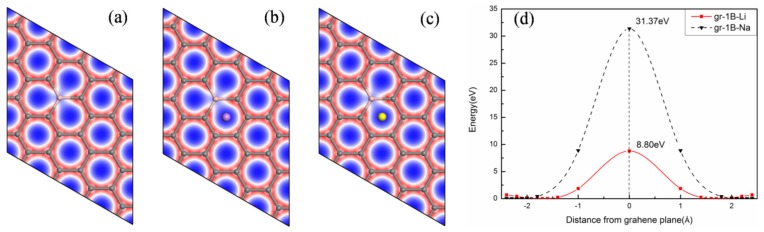
Distribution of the total electronic charge density for B-doped graphene: (**a**) without; (**b**) with Li; (**c**) with Na and (**d**) corresponding diffusion barriers for Li and Na.

Therefore, the effect of B-doping on the barrier for ions to diffuse through the graphene is explored. The adsorption energies of Li and Na for B-doped graphene are −0.95 and −0.74 eV, respectively, as shown in Table 3. Both of them are negative and far below that of 0.49 and 0.61 eV on corresponding original graphene. It shows the optimal stability of the structure of B-doped graphene. As illustrated in Figure 3 (distribution of the total electronic charge density of graphene is plotted in a slab which is parallel to the graphene layer (*xy* plane)), the blue and red filled areas separately means gaining and losing electrons. It demonstrates that there is no charge around Li and B. Because of the electro-positive nature of B, graphene layer receives all its charge. Both Li and Na transfer one electron to the layer as well. The diffusion barriers of Li and Na are 8.80 and 31.37 eV, respectively, as given in Figure 3d. They are slightly lower than that on original graphene. Therefore, the diffusion barriers are not low enough for replacing original graphene by B-doped graphene.

**Table 3:** Adsorption energy (*E_ad_*) of ions, distance of ions from the graphene sheet (*d*) and diffusion barrier (*E_b_*) for the B-doped systems investigated.

System	Li			Na		
	*E_ad_* (eV)	*d* (Å)	*E_b_* (eV)	*E_ad_* (eV)	*d* (Å)	*E_b_* (eV)
Gr-1B	−0.95	1.70	8.80	−0.74	2.20	31.37
Gr-SV-1B	−1.24	1.63	11.01	−0.59	2.20	22.83
Gr-SV-2B	−0.35	1.92	14.43	−0.47	2.30	25.32
Gr-SV-3B	−0.65	1.93	20.02	−0.78	2.30	32.00
Gr-DV-1B	−1.24	1.36	1.95	−1.11	1.90	6.92
Gr-DV-2B(a)	−1.08	1.37	2.27	−0.90	1.91	7.73
Gr-DV-2B(b)	−1.25	1.41	2.41	−1.10	1.91	7.81
Gr-DV-2B(c)	−1.52	1.37	2.25	−1.29	1.90	7.73
Gr-DV-3B	−1.29	1.42	2.64	−1.04	1.95	8.75
Gr-DV-4B	−1.27	1.41	2.86	−1.01	1.97	9.45

**Figure 4:**
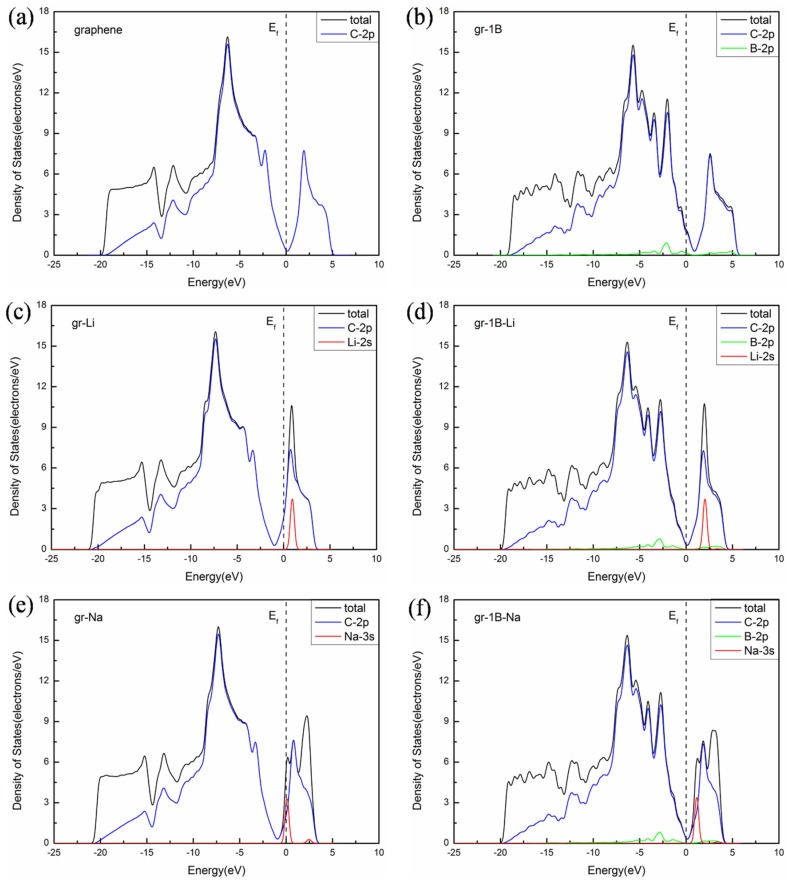
Density of states: (**a**) pristine graphene; (**b**) B-doped graphene; (**c**) pristine graphene with Li; (**d**) B-doped graphene with Li; (**e**) pristine graphene with Na; (**f**) B-doped graphene with Na.

Next, to further discuss the effect of doping on the electronic structure, the calculation of the density of states (DOS) were carried out, as shown in Figure 4. Li and Na are proven to be ionized by the partial density of states (PDOS) of Li-2s and Na-3s. There are peaks of Li-2s and Na-3s above the Fermi level. The PDOS for B-2p shows that B-doping enhances the role of the valence band near the Fermi level. As a result of withdrawing electrons from ions, the diffusion barriers of B-doped graphene exhibit slightly lower than pristine graphene. Furthermore, the positions of the Li-2s and Na-3s peaks above the Fermi level for B-doped graphene are kept slightly higher than that for original graphene. It exhibits the stronger interactions between ions and B-doped system leading to the stronger adsorption energy.

### 3.4. Adsorption and Diffusion of Ions on B-doped Graphene with the Vacancy

**Figure 5:**
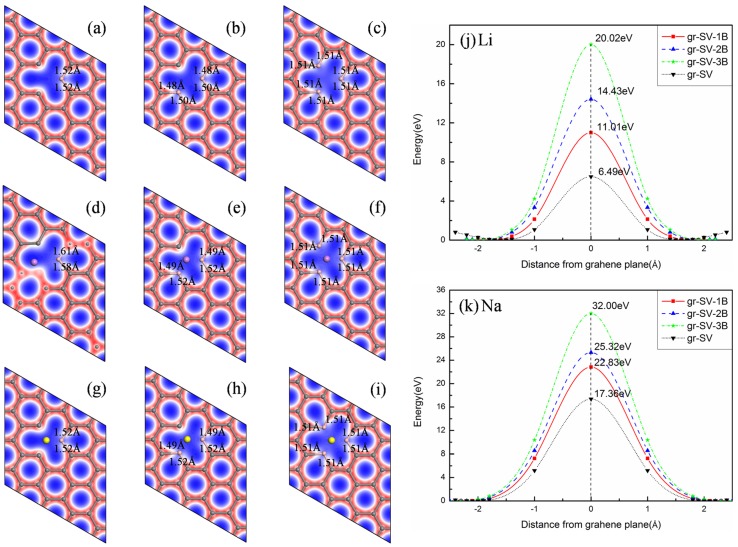
Distribution of the total electronic charge density for graphene with SV doped by: (**a**) one B; (**b**) two B; (**c**) three B; (**d**) one B with Li; (**e**) two B with Li; (**f**) three B with Li; (**g**) one B with Na; (**h**) two B with Na; (**i**) three B with Na; and (**j**) and (**k**) corresponding diffusion barriers for Li and Na.

Boron was doped in the graphene with a single vacancy (SV) and a double vacancy (DV). For the SV defect case, three types of configurations are formed by doping with 1, 2 and 3 boron, as shown in Figure 5a–c. A significant increase of the diffusion barriers for Li (Na) are obtained, owing to the B-doping: (1) 1B doping 11.01 (22.83) eV; (2) 2B doping 14.43 (25.32) eV; and (3) 3B doping 20.02 (32.00) eV, compared to that of 6.50 (17.36) eV in undoped graphene with SV defect, as shown in Figure 5j,k. Such high barriers make the diffusion of Li and Na is almost impossible. Because Li, Na and B are electropositive, there are Coulomb repulsions between them. It is the biggest cause of comparatively reduced adsorption energies of (1) −1.24 (−0.59) eV; (2) −0.35 (−0.47) eV; and (3) −0.65 (−0.78) eV for three kinds of B-doped graphene with SV defect, as illustrated in Table 3. The C-C bond length is 1.389 Å in original graphene [59], and the elongation of C-B bonds are observed significantly in all B-doping structures, as shown in Figure 5a–i. The lengths of the C-B bonds are further extended in the one B-doped system with Li, which buckles particularly. As the result of the repulsion between the Li and B, Li moves to the position 1.63 Å from the plane and keeps away from the center of the defect in this relaxed structure. It provides the highest adsorption energy of −1.24 eV for Li among all the three types of configurations, as shown in Table 3. Li prefers to keep at the distance of 1.92 Å away from the center of the defect in the two B-doped system, which provides the lowest adsorption energy of −0.35 eV for Li. In the case of adsorption of Na, three types of B-doped graphene with SV defect are similar, as shown in Figure 5g–i. In these configurations, Na relaxes at 2.30 Å above the center of the defect, far away from the graphene plane. Hence, Na adsorption has no effect on the C-B bonds in B-doped graphene and the planar structure of that is retained. However, the barriers for Na to diffuse through the defect increase gradually with the increase of the concentrations of B in these configurations. Overall, although the adsorption energies of Li and Na for B-doped graphene with SV are suitable, their diffusion barriers are still very high, which results in that these systems are not advisable as an anode material for ion batteries.

**Figure 6:**
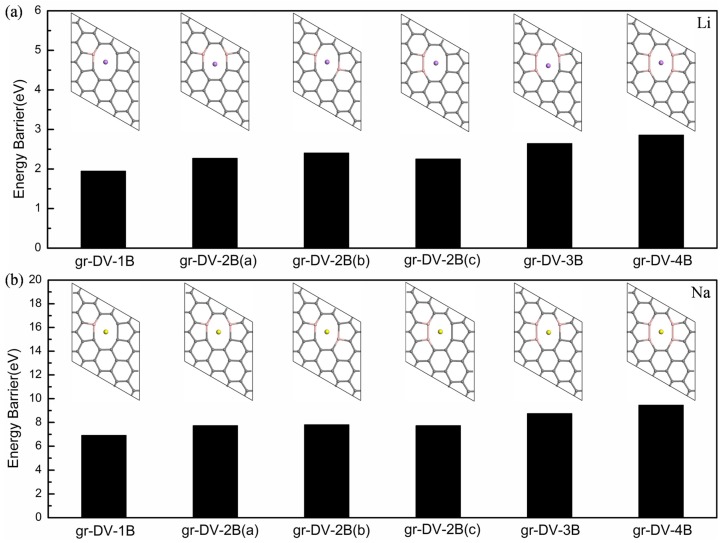
Relaxed structures and corresponding diffusion barriers for B-doped graphene with DV: (**a**) Li and (**b**) Na.

**Figure 7:**
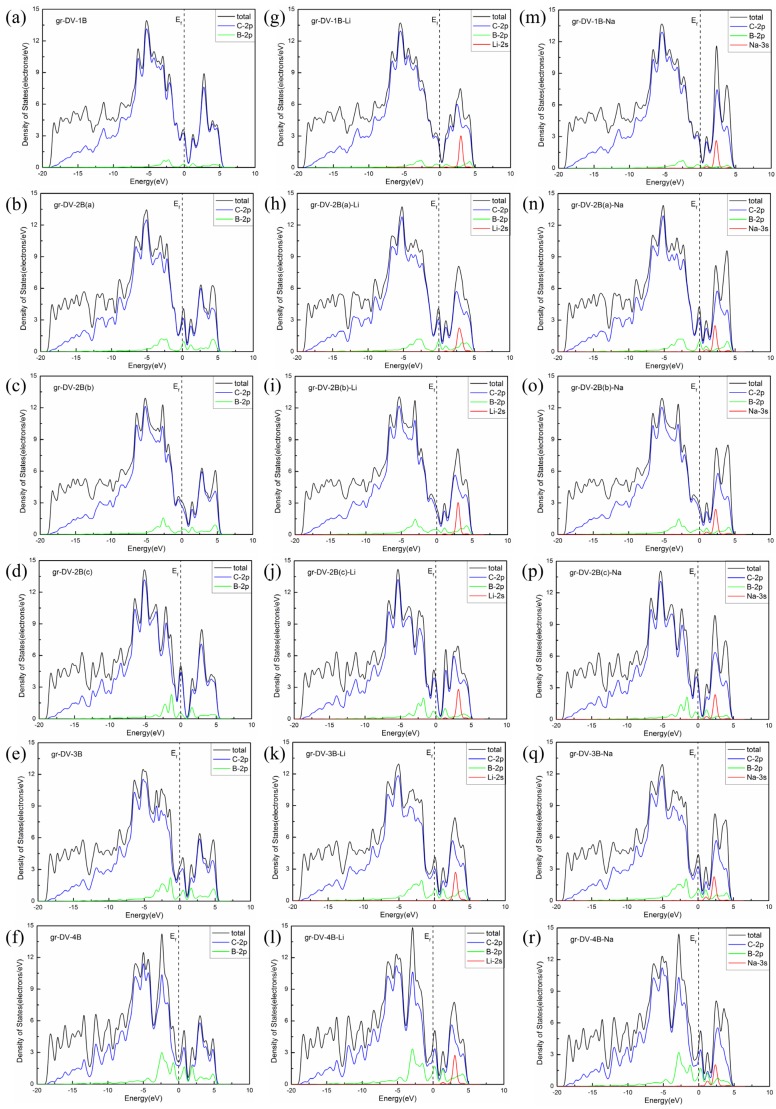
Density of states for graphene with DV doped by: (**a**–**f**) one to four B; (**g**–**l**) one to four B with Li; and (**m**–**r**) one to four B with Na.

Furthermore, we estimate the diffusion barriers of ions through the B-doped graphene with DV. There are six kinds of models for Li and Na diffusion. The structures doped by one to four boron atoms and their corresponding diffusion barriers are shown in Figure 6. The plane structures are almost maintained in all relaxed systems. Both Li and Na are steadily adsorbed above the center of the DV except for the three B doping structure. Because of the large octagon spaces for the ion to diffuse easily, the significant decrease of the barriers can be found. The diffusion barriers of Li and Na for B-doped graphene with DV lie in the range of 1.95 to 2.86 eV and 6.92 to 9.45 eV, respectively. Here, the adsorption energies for Li and Na are also in the optimal range, respectively, from −1.08 to −1.52 eV and from −0.90 to −1.29 eV, as shown in Table 3. The Coulomb repulsions between B and ions contribute to these moderate values of the adsorption energy and the diffusion barrier. Furthermore, the peaks of Li-2s and Na-3s orbitals from the PDOS keep in the same energy range above the Fermi level for all B-doping systems with DV, as shown in Figure 7. It demonstrates that there are similar interactions between ions and B in these systems, further explaining the relatively moderate situation of adsorption energies and diffusion barriers. Thus the B-doped graphene with DV is proved to be a potential anode and SEI candidate base on its low adsorption energies and diffusion barriers.

## 4. Conclusions

We implemented first-principles calculations to clarify the impact of defects and B-doping on the graphene as a possible negative electrode material. Though the barriers for ions are too high to diffuse in pristine graphene, the barriers reduce significantly because of the presence of defects (single vacancy, double vacancy, Stone–Wales defect). It can also be found that the barriers for ions diffusion are determined by the sizes of the open space formed by the defects. Therefore, there are the lowest barriers of 1.49 and 6.08 eV for Li and Na, respectively, in the graphene with DV. The impact of B-doping on the diffusion barriers was also considered. Though the adsorptions of Li and Na on the B-doped graphene are more stable, they are not low enough barriers for fast ion diffusion. Due to the B-doping in the graphene with SV, the high diffusion barriers for Li and Na are observed, which rise gradually with the increase of the concentrations of B in the configurations. However, the B-doped systems with DV provide significantly low adsorption energies and diffusion barriers. Our results show the advantages and disadvantages of various types of graphene, and thus we believe that undoped and B-doped graphene with DV will be better negative electrode and synthetic SEI materials for ion batteries.

## Supplementary Materials

Supplementary materials can be accessed at: http://www.mdpi.com/1996-1944/8/9/6163/s1.
