# Supplementary material for: First-Principles Investigation of Adsorption and Diffusion of Ions on Pristine, Defective and B-doped Graphene
Source: Materials (Basel). 2015 Sep 15;8(9):6163–78. doi: 10.3390/ma8095297 (PMC5512904; doi:10.3390/ma8095297)
Supplement: Supplementary file 1 [file materials-08-05297-s001.pdf]

Supplementary Information

1. Unlike to the pristine graphene, defective graphene are characterized with unsaturated carbon atoms which should be examined more precisely using spin polarized calculation. So we do some test calculations in a few representative systems using spin polarized approach. The results of extra test calculations of migration barriers of ions in defective graphene with a double vacancy (DV) are shown in Figure S1, Tables S1 and S2. All the details of test calculations are consistent with that mentioned in the manuscript, in addition to considering spin polarization. The results of the barriers of ions to diffuse through DV in defective graphene considering spin polarization are shown in Figure S1. The original data set of distances and energies for each step in the diffusion path are provided in Table S1. The energy barriers (E_b) obtained with and without spin polarization for the graphene with DV are compared in Table S2. The migration barriers of ions obtained by using spin polarized calculations are very close to the results ignored spin polarization. Therefore, ignored spin polarization approach is applicative in this paper.

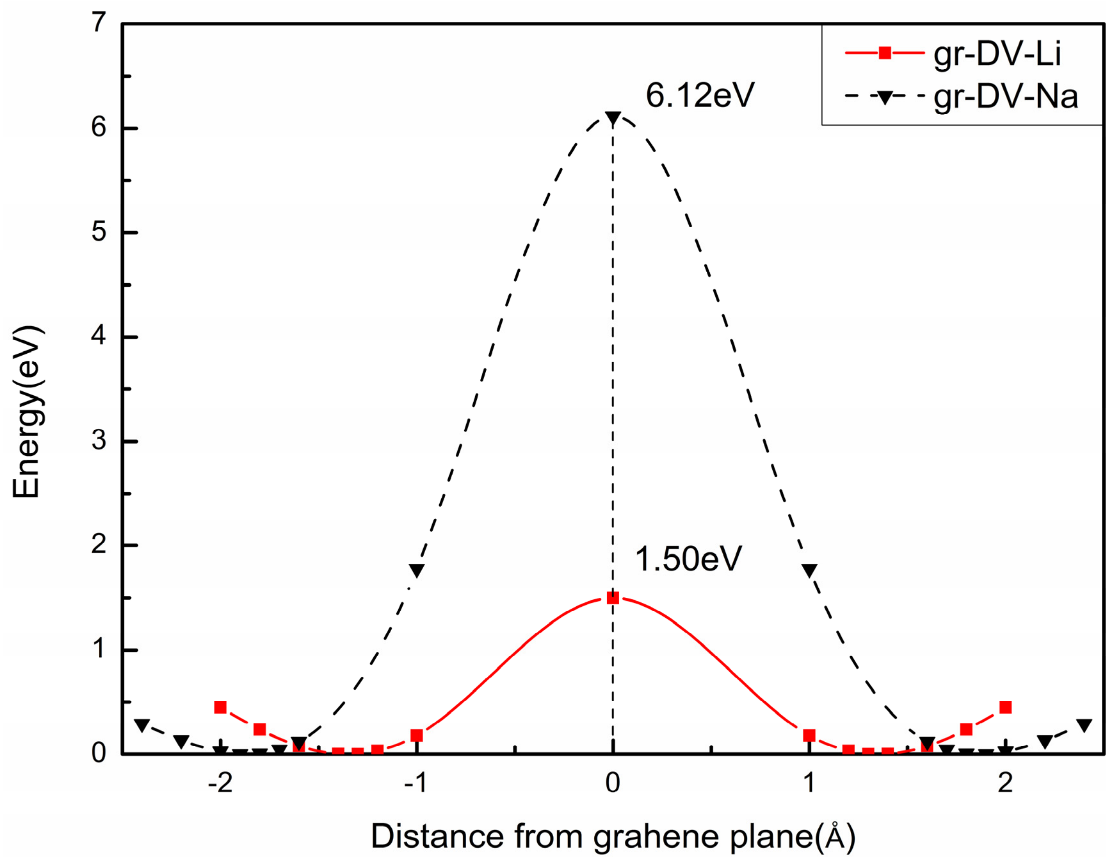

Figure S1. Diffusion barriers for Li and Na to penetrate the graphene with DV via the center the center of the defect.

Table S1. The original data set of distances and energies for each step in the diffusion path.

Gr-DV-Li		Gr-DV-Li (spin polarization)		Gr-DV-Na		Gr-DV-Na (spin polarization)	
d **(Å)**	**Energy (eV)**	***d*** **(Å)**	**Energy (eV)**	***d*** **(Å)**	**Energy (eV)**	***d*** **(Å)**	**Energy (eV)**
/	/	/	/	−2.4	0.289	−2.4	0.291
−2.0	0.445	−2.0	0.447	−2.2	0.133	−2.2	0.133
−1.8	0.234	−1.8	0.236	−2.0	0.025	−2.0	0.025
−1.6	0.077	−1.6	0.078	−1.9	0	−1.9	0
−1.4	0.002	−1.4	0.002	−1.8	0.004	−1.8	0.004
−1.3	0	−1.3	0	−1.7	0.043	−1.7	0.043
−1.2	0.031	−1.2	0.031	−1.6	0.120	−1.6	0.120
−1.0	0.177	−1.0	0.178	−1.0	1.768	−1.0	1.777
0	1.492	0	1.500	0	6.084	0	6.116
1.0	0.177	1.0	0.178	1.0	1.768	1.0	1.777
1.2	0.031	1.2	0.031	1.6	0.120	1.6	0.120
1.3	0	1.3	0	1.7	0.043	1.7	0.043
1.4	0.002	1.4	0.002	1.8	0.004	1.8	0.004
1.6	0.077	1.6	0.078	1.9	0	1.9	0
1.8	0.234	1.8	0.236	2.0	0.025	2.0	0.025
2.0	0.445	2.0	0.447	2.2	0.133	2.2	0.133
/	/	/	/	2.4	0.289	2.4	0.291

Table S2. Comparison of energy barriers (E_b) obtained with and without spin polarization for the graphene with DV.

System	E_b (eV)	E_b (Spin Polarization) (eV)	Error (%)
Gr-DV-Li	1.49	1.50	0.67
Gr-DV-Na	6.08	6.12	0.65

2. To justify the simplified methods adopted for obtaining energy barriers, the verification calculations on a few representative systems, including ions (Li and Na) diffusion through pristine graphene, defective graphene with a double vacancy (DV), and two boron-doped graphene with DV have been implemented using the standard transition state (TS) search combining traditional complete linear and quadratic synchronous transit (LST/QST) algorithm with conjugate gradient method in CASTEP. The diffusion paths of ions moving from one site to the reverse site on the same hexagon, the diffusion barriers, initial structures and transition states are presented in Figure S2. The diffusion barriers obtained by LST/QST method and our simplified approach are compared in Table 1. The relative errors between two methods are less than 5%. Therefore, the results reported here are trustworthy.

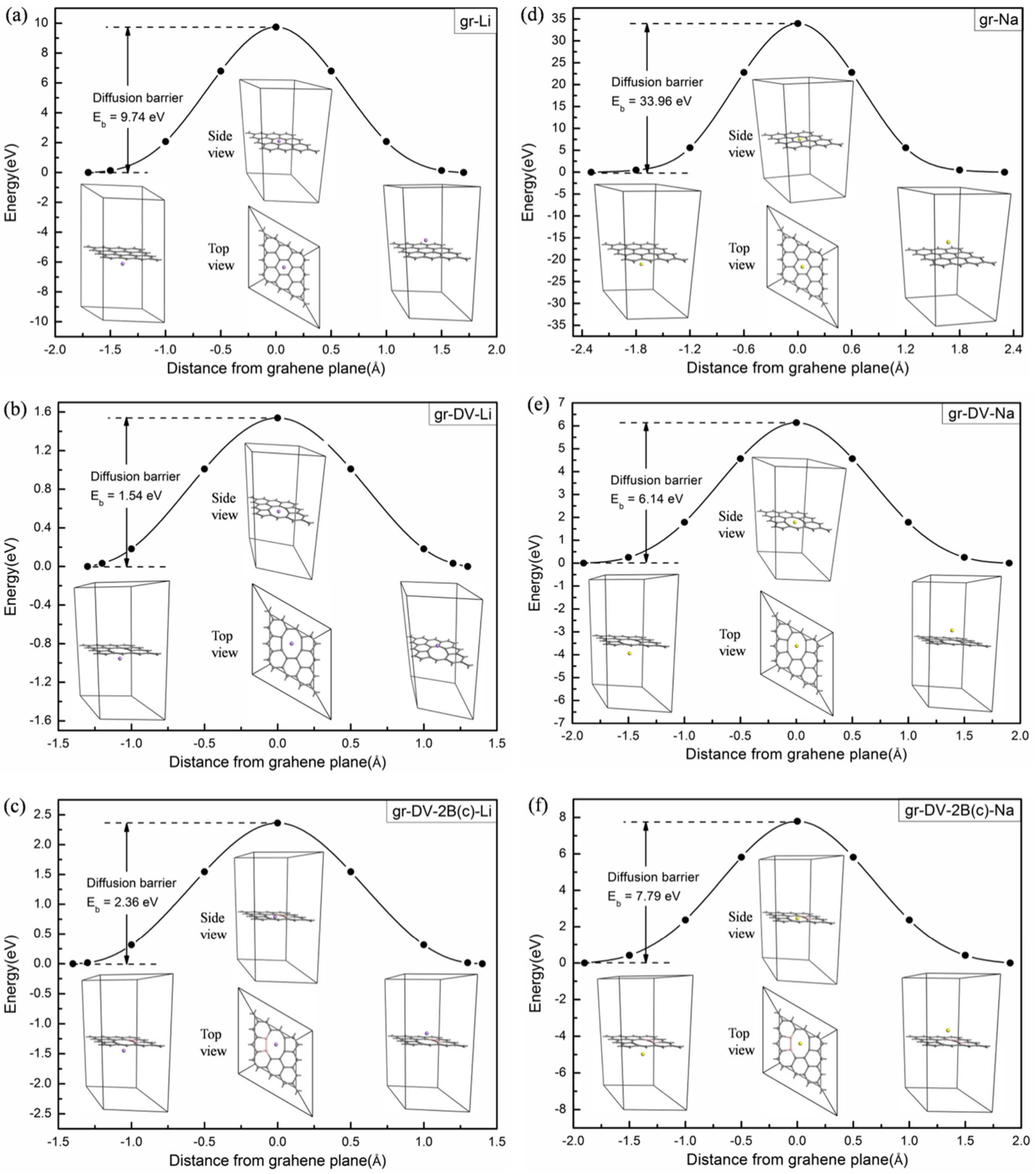

Figure S2. Diffusion barriers along the Li and Na migration path on pristine graphene, defective graphene with DV, and two boron-doped graphene with DV. The diffusion barrier is defined as the energy difference between the transition state and the energy minimum corresponding to the initial state. (**a**) gr-Li; (**b**) gr-DV-Li; (**c**) gr-DV-2B(c)-Li; (**d**) gr-Na; (**e**) gr-DV-Na; (**f**) gr-DV-2B(c)-Na.

3. The original data set of distances and energies for each step in the diffusion path of Li and Na penetrating the pristine and B-doped graphene via the center of the carbon hexagon, corresponding to the line & symbol plots in Figures 1b and 3d.

Table S3. The original data set of distances and energies for each step in the diffusion path of ions penetrating the pristine and B-doped graphene via the center of the carbon hexagon.

**Gr-Li**		**Gr-Na**		**Gr-1B-Li**		**Gr-1B-Na**	
d **(Å)**	**Energy (eV)**	***d*** **(Å)**	**Energy (eV)**	***d*** **(Å)**	**Energy (eV)**	***d*** **(Å)**	**Energy (eV)**
−2.1	0.264	−2.8	0.127	/	/	/	/
−2.0	0.159	−2.5	0.032	−2.4	0.697	/	/
−1.9	0.070	−2.4	0.009	−2.2	0.418	−2.4	0.085
−1.8	0.016	−2.3	0	−2.0	0.181	−2.3	0.029
−1.7	0	−2.2	0.008	−1.8	0.024	−2.2	0
−1.6	0.030	−2.1	0.045	−1.7	0	−2.1	0.011
−1.5	0.131	−1.9	0.262	−1.6	0.019	−1.9	0.076
−1.3	0.572	−1.7	0.852	−1.4	0.258	−1.7	0.429
−1.0	2.065	−1.2	5.590	−1.0	1.858	−1.2	8.887
0	9.707	0	33.941	0	8.796	0	31.374
1.0	2.065	1.2	5.590	1.0	1.858	1.2	8.887
1.3	0.572	1.7	0.852	1.4	0.258	1.7	0.429
1.5	0.131	1.9	0.262	1.6	0.019	1.9	0.076
1.6	0.303	2.1	0.045	1.7	0	2.1	0.011
1.7	0	2.2	0.008	1.8	0.024	2.2	0
1.8	0.016	2.3	0	2.0	0.181	2.3	0.029
1.9	0.070	2.4	0.009	2.2	0.418	2.4	0.085
2.0	0.159	2.5	0.032	2.4	0.697	/	/
2.1	0.264	2.8	0.127	/	/	/	/
